# Surface Functionalization of Cellulose-Based Packaging with a New Antimicrobial Decapeptide: A Sustainable Solution to Improve the Quality of Meat Products

**DOI:** 10.3390/foods14152607

**Published:** 2025-07-24

**Authors:** Bruna Agrillo, Rosa Luisa Ambrosio, Valeria Vuoso, Emanuela Galatola, Marta Gogliettino, Monica Ambrosio, Rosarita Tatè, Aniello Anastasio, Gianna Palmieri

**Affiliations:** 1 Institute of Biosciences and BioResources (IBBR), National Research Council (CNR), 80131 Naples, Italy; bruna.agrillo@cnr.it (B.A.); emanuela.galatola@ibbr.cnr.it (E.G.); marta.gogliettino@cnr.it (M.G.); monica.ambrosio@ibbr.cnr.it (M.A.); 2 Department of Veterinary Medicine and Animal Production, University of Naples Federico II, 80137 Naples, Italy; rosaluisa.ambrosio@unina.it (R.L.A.); valeria.vuoso@unina.it (V.V.); 3 Institute of Genetics and Biophysics (IGB), National Research Council (CNR), 80131 Naples, Italy; rosarita.tate@cnr.it

**Keywords:** cellulose-based packaging, antimicrobial peptides, food safety, shelf life, beef carpaccio

## Abstract

The need for renewable and eco-friendly materials is driving the increasing demand for biobased polymers for food applications, with cellulose emerging as a promising option due to its degradability and environmental sustainability. Therefore, in the present study, a strategy to obtain cellulose-based materials with antimicrobial properties was explored by using a selected antimicrobial peptide named RKT1, which was stably and efficiently tethered to cellulose films via physical adsorption, harnessing the high number of functional groups on the polymeric surface. Firstly, the peptide, identified among the previous or new projected compounds, was structurally and functionally characterized, evidencing high conformational stability under a wide range of environmental conditions and efficient antibacterial activity against the foodborne pathogens *Escherichia coli*, *Salmonella* Typhimurium, and *Listeria monocytogenes* and the spoilage bacteria *Enterococcus* and *Pseudomonas koreensis,* all isolated from meat products. Moreover, in an extended application, the RKT1-activated cellulose films were tested in vivo on beef carpaccio. The results supported their effectiveness in increasing the shelf life of carpaccio by least two days without affecting its organoleptic properties. Therefore, RKT1, physically adsorbed on cellulose, still retains its activity, and the newly generated biopolymers show potential for use as a green food packaging material.

## 1. Introduction

Active packaging plays a critical role in enhancing food quality by extending shelf life, maintaining freshness, and reducing spoilage. Unlike passive packaging, which serves as a barrier, active material interacts with the food products or their environments. This technology allows substances to release or absorb moisture, oxygen, or other elements, thereby preventing microbial growth and delaying the oxidation of food components, which strongly affects food quality. As a result, the products remain fresh longer, the nutritional values are preserved, and the overall safety is improved. This innovative approach is beneficial specifically for perishable items such as fruits, vegetables, dairy, and meat, ensuring optimal conditions from production to consumption.

Nowadays, plastic materials with long-lasting stability, such as polyethylene (PE), polystyrene (PS), or polypropylene (PP) [1,2], are the most frequent supplies used for applications in active packaging for food products with a short shelf life. However, most plastic materials are single-use and cannot be recycled, causing severe environmental impacts. In addition, the use of traditional petroleum-derived plastics can pose potential food hazards, environmental pollution, and human health concerns due to the incineration or landfilling of waste, as well as the possible release of plastic compounds or functional additives [3,4,5]. It has been estimated that approximately 500 billion plastic bags are used annually worldwide [6].

Therefore, the increasing demand for new food packaging that is environmentally friendly, renewable, and sustainable has strongly increased research activities over the last decades. In this contest, biobased materials, such as polysaccharides [7,8,9], polylactic acid (PLA), and polyhydroxyalkanoate (PHA) [10,11,12], have emerged as one of the most promising innovations, showing remarkable potential due to their environmental friendliness and sustainability. Among these bioresources, cellulose can represent a valuable solution for packaging applications, considering the need for both economic and environmental benefits [13,14]. However, although cellulose in its natural form offers some advantages, such as biodegradability, availability, and renewability [14,15], it has several limitations, including the inherent lack of antimicrobial activity and potential for microbial contamination during processing. This restricts its commercial use specifically in applications where such activity is desired, like food packaging. Therefore, to overcome this, cellulose films need to be modified with antimicrobial agents, thus acquiring antibacterial performance, which ensures food protection and prevents foodborne illnesses [3,4].

In this scenario, a significant role could be played by the antimicrobial peptides (AMPs), which are naturally occurring compounds produced by all living organisms, showing a broad-spectrum antimicrobial activity against bacteria, fungi, and viruses [16,17,18]. In mammals, AMPs such as defensins are key components of the innate immune system that are able to counteract microbial infections and prevent the establishment of pathogens [16,17,18]. AMPs are generally classified according to their source of origin, action, structure, and amino acid composition. They are mostly cationic monomers of 4–50 amino acids showing a strong tendency to form amphipathic secondary structures of α-helix, β-hairpin-like β-sheet, β-sheet, or α-helix/β-sheet mixed structures [19,20,21]. These physicochemical characteristics improve the AMPs’ ability to interact with negatively charged microbial membranes. The principal mechanism reported for AMPs is associated with their capacity to lyse microbial cells, as the cationic properties (net positive charge) of most of these compounds allow them to efficiently interact with the negatively charged membranes of microorganisms. However, further mechanisms have been described in which AMPs target specific intracellular target molecules [19,20,21].

AMPs have potential applications in the food packaging industry, but they must fulfil several criteria to function as efficient coating agents on different material surfaces, including the retention of broad-spectrum antimicrobial activity in the tethered state. In addition, the stability and functional activity of these “activated” materials under various environmental conditions still remain underexplored. Many naturally occurring peptides do not maintain their properties, necessitating the development of new and more effective AMPs to enhance the safety and shelf life of food products.

In this study, starting from the previously characterized antimicrobial peptide RiLK1 [22,23,24], a new decapeptide named RKT1 was rationally designed based on the modification of selected amino acids in the polypeptide chain backbone, and the relevant physicochemical properties were predicted using different bioinformatics tools. RKT1 was functionally and structurally characterized, revealing high stability in a wide range of pH and temperature for prolonged incubation times and a broad spectrum of antibacterial activity against some of the most important foodborne pathogens and spoilage bacteria. Therefore, the efficacy of RKT-associated cellulose materials was investigated to elucidate the functional properties and effectiveness in improving food safety and shelf life and reducing waste.

The findings of this research are expected to provide insights into the development of innovative packaging solutions that combine sustainability with enhanced antimicrobial protection. This could significantly benefit industries reliant on prolonged shelf life and stringent hygiene standards, thereby contributing to reduced food spoilage and improved consumer safety.

## 2. Materials and Methods

### 2.1. Synthesis and In Silico Design of RKT1

The peptide synthesis of RKT1 (RKTWLRIWKR-NH2) was performed at GenScript Biotech (Leiden, The Netherlands) and achieved with a high level of purity (>95%). The freeze-dried peptide powder was stored at −20 °C. Analysis by mass spectrometry confirmed the identity of the peptide. The analysis of all the main physical and chemical characteristics of RKT1 was determined using the following web servers and software: PlifePred (PPred) [25], PEPlife [26], and Antimicrobial Peptide Database3 (APD3) [27].

### 2.2. Circular Dichroism Spectroscopy

Circular dichroism (CD) analysis was carried out through the Jasco J-810 (Jasco, Tokyo, Japan) spectropolarimeter using a quartz cuvette of a 0.1 cm path length (Hellma Analytics). All the spectra were recorded in the 195 nm–250 nm range at a scan speed of 20 nm/min, averaged over 5 scans. CD spectra of RKT1 (50 µM) were acquired with SDS at three different concentrations (3, 50 and 150 mM) in Tris-HCl 10 mM pH 7.0. The effects of pH on the secondary structural elements of RKT1 were assessed by dissolving 50 μM of the peptide in different buffer solutions (10 mM glycine-HCl, pH 2.0; 10 mM Tris-HCl, pH 7.0; 10 mM glycine-NaOH, pH 11.0) and adding SDS to a final concentration of 50 mM. Analysis of the prepared samples was carried out after 48 h of incubation at 25 °C. For thermal stability, the same quantity of peptide was incubated in 10 mM Tris-HCl pH 7.0 and kept at 4, 37, and 90 °C up to 48 h before adding 50 mM SDS and acquiring the CD spectra. The analysis of peptide folding kinetics (50 µM in Tris-HCl 10 mM pH 7.0) was conducted up to 24 h by adding a micellar concentration of SDS (50 mM) to each sample. The mean residue ellipticity value ([θ], deg cm^2^ dmol^−1^) was calculated considering the peptide concentration (mM), the number of amino acid residues, and the cell path length (cm). For each measure, the background (buffer) was subtracted from the signal.

### 2.3. Fluorescence Spectroscopy

Fluorescence analyses (folding kinetics, effect of different SDS concentrations and pH on peptide folding and thermal stability) were carried out on a Shimadzu RF-6000 spectrofluorometer (Kyoto, Japan) under the same experimental conditions described above. Emission spectra of tryptophan were recorded at 25 °C by setting the slit widths to 5 nm. The intrinsic tryptophan was excited at a wavelength of 280 nm, and the emission was monitored between 300 and 400 nm. The folding kinetic experiments of RKT1 were carried out after the addition of SDS (50 mM) to each sample (50 μM concentration in 10 mM Tris-HCl, pH 7.0), up to 24 h of incubation. The effect of pH on peptide folding was evaluated by dissolving the peptide at a final concentration of 50 μM in buffer at pH 2.0, 7.0, or 11.0. Then, the SDS (50 mM final concentration) was added to each sample, which was incubated up to 48 h at 25 °C and analyzed by fluorescence spectroscopy. For thermal stability, the peptides were prepared to a final concentration of 50 M in 10 mM Tris-HCl buffer, pH 7.0, in the presence of 50 mM SDS, and then they were incubated at 4, 37, and 90 °C up to 48 h.

### 2.4. RKT1 Adsorption on Cellulose Film Surfaces

Commercial cellulose (CI) films, typically used for the conservation of meat products, were kindly provided by Visioni Srl (Naples, Italy). For in vitro tests, individual CI films were cut into slides of 6 cm^2^ and incubated with the minimum volume (1 mL) of peptide solution (50 µM concentration) or water at 60 °C until the aqueous solution was completely removed (50 min), thus favoring the adsorption of the molecules on the surface. After drying, the functionalized slides were wetted with 1 mL of distilled water and sonicated in a Bandelin-SONOREX SUPER RK 100 H, for 15 min with an ultrasonic frequency of 35 kHz at 25 °C. Therefore, the recovered solutions (1 mL for each sample) were centrifuged at 12,000 rpm and analyzed by reverse-phase high-performance liquid chromatography (RP-HPLC) to calculate the immobilization yield of the peptide, indirectly. To improve antimicrobial efficacy, the slides were functionalized by immobilizing the peptide twice on the same side, applying the procedure previously described after drying the films under a nitrogen flow.

### 2.5. Immobilization Yield Analysis

The functionalization yield of the bound RKT1 was assessed by RP-HPLC. After each reaction, 200 μL of the recovered solutions (1 mL for each slide) was loaded onto a μBondapak reversed-phase C18 column (Waters, Milford, MA, USA, 3.9 mm × 300 mm) connected to an HPLC system (Shimadzu, Milan, Italy), using a 19 min linear gradient of 0.1% TFA in acetonitrile (5–95%). A reference solution with the same peptide concentration used for immobilization reactions was analyzed. Therefore, the percentage of RKT1 adsorbed onto the surface was indirectly calculated by comparing the peak area with that of the reference solution at the end of the functionalization. A calibration curve using different RKT1 concentrations (ranging from 25 µM to 200 µM) was built. The standard curve displayed a strong linear relationship between peak area and peptide concentrations with a correlation coefficient (R^2^) of 0.9983. All measurements were performed in triplicate on three different preparations. Moreover, the amount of adsorbed RKT1 on the cellulose films was determined by using a Radwag MYA-5.4Y analytical balance (Radom, Poland) as the difference in weight between pristine and treated CI films [28]. The statistical significance of differences between the samples in the presence or absence of a peptide was calculated using the Student’s *t*-test, with a significance level of *p* < 0.05.

### 2.6. Stereomicroscopy

Small pieces of cellulose films, untreated or treated twice with RKT1 (50 μM for each adsorption step) were placed in Petri dishes and stained with a 0.1% Ponceau Red solution in 5% acetic acid at room temperature for 10 min. After the incubation time, the cellulose pieces were transferred to Petri dishes and washed three times with 5% acetic acid. Therefore, the pieces were placed on a microscope slide and covered with a cover slip. After drying overnight, the samples were observed under a Leica MZ16FA (Wetzlar, Germany) stereomicroscope and photographed.

### 2.7. Release Test

To verify the stability of RKT1 on the functionalized polymers, a release assay was conducted by RP-HPLC using the same experimental protocol described in the “Immobilization yield analysis” paragraph. After the two-step immobilization process, the dried functionalized polymers were dipped into a 1% NaCl solution (1 mL for each sample) and incubated for 7 days at 25 °C. At the end of different incubation times, the salt solution was recovered and sonicated in a Bandelin-SONOREX SUPER RK 100 H (Milan, Italy), for 20 min with an ultrasonic frequency of 35 kHz at 25 °C. Then, 200 μL was withdrawn and loaded on an RP-HPLC C18 column. Unfunctionalized CI films were used as a control. The concentration of the peptide from the films was calculated using the calibration curve, that was built using different RKT1 concentrations (ranging from 25 µM to 200 µM). The standard curve displayed a strong linear relationship between the peak area and peptide concentrations with a correlation coefficient (R^2^) of 0.9983. Therefore, the percentage of release was determined with respect to the nmol/cm^2^ of the adsorbed peptide. All analyses were repeated three times.

### 2.8. In Vitro Antimicrobial Activities of RKT1

To evaluate the antimicrobial activities of RKT1, both pathogenic and specific spoilage bacteria, previously isolated from foods, were used. Specifically, pathogenic strains of *Listeria monocytogenes*, *Salmonella* Typhimurium, and *Escherichia coli* were provided by the Laboratory of the Department of Food Microbiology of the Istituto Zooprofilattico Sperimentale del Mezzogiorno in Portici (Naples, Italy), while spoilage bacteria *Enterococcus* spp. and *Pseudomonas koreensis* were isolated from meat and meat products in the Food Inspection Laboratory of the Department of Veterinary Medicine and Animal Production of the University of Naples “Federico II”, and identified with the MALDI-TOF mass spectrometry technique. Minimum inhibitory concentration (MIC) and minimum bactericidal concentration (MBC) were determined by the standard broth serial dilution method following the Clinical & Laboratory Standards Institute guidelines (CLSI,) [29]. MIC is defined as the lowest concentration of the peptide that inhibits bacterial growth, while MBC is defined as the lowest concentration of the peptide at which more than 99.9% of the bacterial cells are killed. For the microbroth dilution assay, all bacteria were grown in buffered peptone water (BPW) (Thermo Fisher, Milan, Italy). Growth of bacterial cells was achieved by incubating them at 37 °C in specific culture media, then the working concentration was obtained by diluting fresh broth to a final concentration of 1.0 × 10^5^ CFU/mL (CFU, colony forming units) for the pathogenic bacteria and 1.0 × 10^3^ CFU/mL for the spoilage. Thereafter, different concentrations of RKT1 were added to each bacterial suspension to obtain final peptide concentrations ranging from 3 to 50 µM. Control samples, consisting of cell suspensions only, were included. Samples were incubated at 37 °C for 6 h. MBC was determined by plating 50 µL of each bacterial cell suspension on selective agar: L. *monocytogenes* on Agar Listeria according to Ottaviani & Agosti (ALOA) (Biolife, Italy); *S*. Typhimurium on Salmonella Chromogenic agar (Oxoid, UK); *E. coli* on TBX agar (Biolife, Italy); and *Enterococcus* spp. and *P. koreensis* on plate count agar (PCA) (Oxoid, UK). Plates were incubated at 37 °C for 24–48 h, except for *E. coli*, which was incubated overnight at 44 °C. The data are representative of three independent experiments performed in triplicate.

### 2.9. In Vitro Antimicrobial Activities of Functionalized Cellulose Slides

The antimicrobial activities of functionalized cellulose have been tested in vitro using a modified version of the microdilution broth assay [30,31,32,33,34]. For the tests, two bacterial strains, *E. coli* and *P. koreensis*, collected from meat and meat products, were used. Briefly, bacterial suspensions of approximately 3.5 log (CFU/mL) in tryptone soy broth (TSB; OXOID, Madrid) were prepared from revitalized frozen stocks. Aseptically, cellulose films, functionalized (CI-RKT1) or not (CI-CTR) with the peptide, were placed at the bottom of a glass tube and immersed in 10 mL of bacterial suspension. The tubes were incubated at specific temperatures (25 °C for *P. koreensis* and 37 °C for *E. coli*) and kept under horizontal agitation (160 rpm; HS260 Basic IKA). At 3, 6, and 24 h of incubation, 100 μL of bacterial suspensions were plated directly on an agar medium specific to *E. coli* (TBX agar) and *P. koreensis* (Pseudomonas agar base). In addition, tenfold serial dilutions were prepared from each tube for enumeration. The plates were incubated for 24 h before the final enumeration. The experimental tests were performed as previously reported [30,31,32,33,34], using cellulose films with an area of approximately 6 cm^2^. The experiments were repeated three times.

The antimicrobial activity was calculated as “bacterial reduction” caused by the presence of the antimicrobial peptide on the cellulose surface [32], following the formula:

log (CFU/mL)_CI-CTR_–log (CFU/mL)_CI-RKT1_

### 2.10. Shelf Life Studies on Packed Beef Carpaccio

#### 2.10.1. Samples Preparation

Shelf life studies were carried out on beef carpaccio to evaluate the effect of active packaging on its “life”. Specifically, to validate the obtained results, the beef carpaccio samples enrolled for analysis belonged to two different batches (trail I and trial II). For each batch, the samples were split into two experimental groups: a control group (CI-CTR), including beef carpaccio packaged in non-functionalized CI films (cbCTR, nine samples per batch), and a treated group (CI-RKT1), comprising beef carpaccio packaged with CI films functionalized with RKT1 (cbRKT1, nine samples per batch). According to the analytical protocol, upon the arrival of the samples in the laboratory, the beef carpaccio packages were opened to put the functionalized and non-functionalized cellulose sheets in contact with the samples. The packages were closed using food cling wrap, paying attention to avoiding opening points that would have allowed microbiological contamination from the external environment. Then, the packed samples were stored at 4 ± 1 °C for 7 days. Microbiological and physicochemical analyses, as well as sensory evaluation, were carried out in triplicate at days 0, 4, and 7.

#### 2.10.2. Microbiological Analysis

To thoroughly evaluate the potential antimicrobial activity of CI-RKT1 on beef carpaccio, viable counts of various microorganisms were performed. The protocols adopted were those recognized by the International Organization for Standardization (ISO). Specifically, the concentrations of the following bacteria were evaluated: Total mesophilic bacterial load (TMB, ISO 4833-1:2013), *Enterobacteriaceae* (ISO 21528-2:2017), *Pseudomonas* sp. (ISO 13720:2010); *E. coli* beta-glucuronidase-positive (ISO 16649-1:2018), mesophilic lactic acid bacteria (LAB, ISO 15214:2015), yeasts and molds (ISO 21527:2008), *Brochothrix* sp. (ISO 13722:2017), and coagulase-positive *staphylococci* (ISO 6888-1:2021). After counting, the data were expressed as logarithms of the number of colony-forming units (CFU/g), and means and standard error were calculated.

#### 2.10.3. Chemical and Physical Analysis

For both batches, water activity (aw) was monitored with Aqualab 4 TE (Decagon Devices Inc., Pullman, WA, USA) as well as lipid peroxidation by measuring the concentration (mg/kg) of malondialdehyde (MDA; CH_2_(CHO)_2_), which is recognized as a marker of secondary oxidation. This analysis was performed according to the method proposed by Ambrosio et al. [35].

#### 2.10.4. Changes in the Color of Packed Beef Carpaccio

Finally, an objective sensory evaluation was carried out to highlight differences in the color appearance of samples packaged with functionalized cellulose (CI-RKT1).

Colorimetric measurements of the beef carpaccio surface were performed using a Konica Minolta CR 300 colorimeter (Minolta, Osaka, Japan). The data were analyzed in the CIELAB color space, organized in three orthogonal axes in a Cartesian coordinate system: lightness (L*), redness (a*), and yellowness (b*). Specifically, total color difference (ΔE) and variation in a* (Δa*) were calculated according to Equation (1):

(1) $$\Delta E\;=\;\sqrt{{\left({L^{*}}_{1}-{L^{*}}_{2}\right)}^{2}+{\left({a^{*}}_{1}-{a^{*}}_{2}\right)}^{2}+{\left({b^{*}}_{1}-{b^{*}}_{2}\right)}^{2}}$$

$$\Delta a*={a^{*}}_{2}-{a^{*}}_{1}$$

where L*_2_, and a*_2_ are the values obtained at day 4 or day 7, during the storage; instead, L*_1_ and a*_1_, are those collected at day 0.

To obtain representative results and avoid using data contaminated by normal color differences that can be found on the surface of beef carpaccio, several measurements were performed on three different areas of the surface of each sample.

### 2.11. Statistical Analysis

Analyses of variance were performed using Prism v. 10.0 (GraphPad Software Inc., San Diego, CA, USA). Microbiological results, physicochemical data and color measurements were compared between control and treated groups using a 2-way ANOVA followed by Tukey’s multiple comparisons test or Holm-Šídák multiple comparisons test, to identify significant differences between pairs (*p* < 0.05). Furthermore, three-way ANOVA was performed to calculate the effects of each ANOVA factor (days of storage, type of packaging, and batch of carpaccio).

## 3. Results

### 3.1. Selection of Antimicrobial Peptides to Develop Antimicrobial Cellulose-Based Films

The meat industry is concerned about the rise in foodborne diseases caused by eating contaminated food, which result in significant economic losses. In this context, antimicrobial packaging represents a valuable and excellent option for slowing down microbial growth, thereby extending shelf life while preserving meat quality during storage. However, most widely used packaging materials are non-biodegradable plastics, thus posing significant environmental and health risks. Therefore, the food industry is increasingly turning to more sustainable alternatives, such as biodegradable and renewable materials.

In this work, cellulose film, one of the materials commonly used in the food industry, especially by fast food chains [14,15], was chosen as a model biopolymer to develop a new class of antimicrobial packaging. In our previous studies, both the structural and functional aspects of a panel of projected peptides named 1018-K6 [36], MTP1 [37], RiLK1 [22,23,24], RiLK3 [22,24], and RiLK30 [23] were thoroughly explored, thus leading to the current investigations on the potential use of these compounds to functionalize the surfaces of cellulose films. Moreover, to expand the portfolio of molecules with antimicrobial activity against bacteria associated with food spoilage and/or food poisoning, a set of novel small peptides was designed using a rational approach. In detail, specific and single-point amino acid substitutions were introduced in the sequence of the decapeptide RiLK1, chosen as the lead AMP due to its broad spectrum of antimicrobial activity and low production costs, taking into account the importance of preserving all the structural features able to promote membrane interactions and lead to increased antimicrobial activity, such as a balance of positive charges, overall hydrophobicity, and the presence of lipophilic residues like Tryptophan (Trp). Specifically, the projected strategy involved the combined and integrated use of different software packages [25,26,27], among those most frequently applied for in silico analysis of peptide sequences. This approach enables accurate determination of the physicochemical properties, which are important for the adopted predictive process and generally associated with the peptide’s biological function. Therefore, a 10-mer peptide, named RKT1, was designed and selected, following the construction of an ad hoc decision tree model, which was applied starting from the sequence of RiLK1. The developed model comprises a series of “decision nodes,” each representing a “test” on one of the eight identified physicochemical parameters (Bomax index, net charge, half-life, hydrophobicity, hydropathicity, amphipathicity, hydrophilicity, and secondary structure). To choose among alternatives and optimize the decision quality, criteria such as threshold limits were used to evaluate and identify those candidates that led to the definition of the most promising peptide of this study in terms of potential antimicrobial activity, high stability, non-cytotoxicity.

Based on this approach (Table 1), the de novo peptide RKT1 was selected, together with the compounds already characterized, as the suitable candidates to be used in the surface’s immobilization procedures, performed through the adsorption of the bioactive compounds on polymeric surfaces, to develop antimicrobial cellulose films.

This was achieved by exploiting the physical characteristics of this material, which provides a surface that can readily interact with peptides via hydrogen bonds and van der Waals forces, due to its abundant polar and hydrophobic groups. It is worth noting that the physical absorption process was chosen among the many different methods, such as covalent binding, entrapment, or cross-linking [38], because it is the simplest one, and it can be carried out under mild conditions, being based on physical interactions [39].

The success of the developed procedure was assessed by reverse-phase high-precision liquid chromatography (RP-HPLC), analyzing the supernatant solutions recovered after rinsing the films with water. Specifically, the amount of peptide absorbed on CI films was indirectly determined using the method described in Section 2.5. The results showed that only RKT1, among all the peptides tested, was able to efficiently adsorb on cellulose materials, reaching the 100% immobilization yield (Figure S1). Therefore, it was selected as the model compound to proceed with further analyses.

### 3.2. Structural Characterization of RKT1

The diversity of the secondary structures adopted by AMPs represents one of the most fundamental features which strongly influence their ability to interact with bacterial membranes and disrupt them, ultimately enabling peptides to exhibit antimicrobial effects. Therefore, the secondary structure of RTK1 in a membrane-like environment was assessed by circular dichroism (CD) spectroscopy, dissolving the peptide (50 µM) in increasing concentrations of SDS, leading to the formation of micelles.

**Figure 1:**
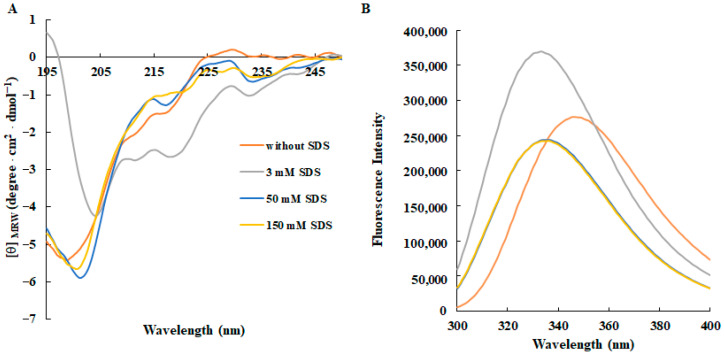
Effects of SDS concentration on the secondary structure and conformation of RKT1 monitored by spectroscopic techniques. (**A**) CD spectra and (**B**) fluorescence emission spectra of RKT1. All spectra were recorded at 25 °C using a peptide concentration of 50 μM in 10 mM Tris-HCl buffer, pH 7.0 and in the absence (orange lines) or in the presence of SDS at different concentrations.

As shown in the CD spectra reported in Figure 1A, the RKT1 signal minimum shifted from 198 to 205 nm with a concomitant decrease in the CD signal at increasing concentration of SDS, correlating with the transition from unordered structures (random coil) in the absence of SDS to self-assembled states in which the peptide spontaneously forms ordered aggregates, as already observed with its parental analogous [22,23,24].

Then, the presence of Trp residues in the peptide sequence allowed us to probe for changes in their environment by examining SDS-induced shifts occurring in the wavelength of their fluorescence emission maximum. In an aqueous solution, the emission maximum for the peptide was ∼350 nm, but upon addition of SDS at a 3 mM concentration, a blue shift in the λmax from 350 to 335 nm occurred, indicative of the ‘moving’ of Trps to a polar environment, accompanied by the characteristic increase in intensity. Above the critical micellar concentration (CMC), the intensity of the fluorescence emission peaks decreased, suggesting a strong insertion of Trp residues into the hydrophobic core of the SDS acyl chains, resulting in their shielding (Figure 1B). However, a self-quenching process could take place upon binding with micelles due to peptide oligomerization, as also confirmed by CD spectra.

**Figure 2:**
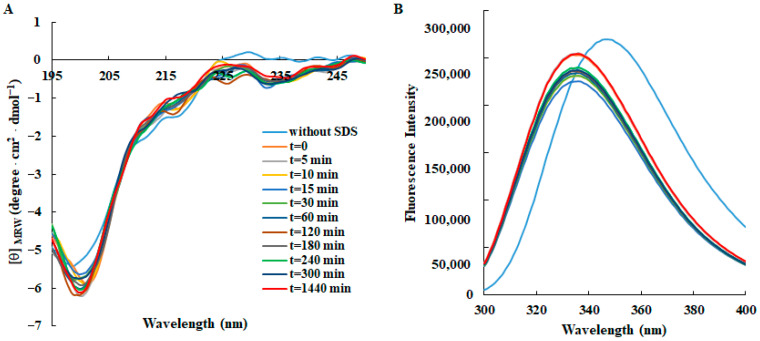
Time-dependent effect of SDS on the secondary structure and conformation of RKT1 monitored by spectroscopic techniques. (**A**) CD spectra and (**B**) fluorescence emission spectra of RKT1. All spectra were recorded at a peptide concentration of 50 μM in 10 mM Tris-HCl, pH 7.0, in the absence (blue lines) or in the presence of SDS (50 mM) up to 24 h incubation at 25 °C.

Moreover, the folding kinetic of RKT1 in the presence of 50 mM SDS during the 24 h incubation was studied. Both the CD (Figure 2A) and fluorescence spectra (Figure 2B) evidenced that the peptide did not undergo significant conformational change during the folding process.

**Figure 3:**
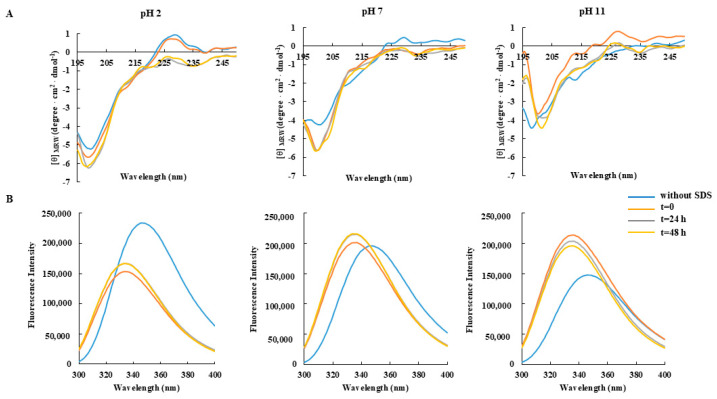
Effect of pH on the secondary structure and conformation of RKT1. (**A**) CD spectra and (**B**) fluorescence emission spectra of RKT1. All spectra were obtained by incubating the peptide (50 μM) in buffers at different pHs for 48 h at 25 °C, and in the presence of SDS at a final concentration of 50 mM.

**Figure 4:**
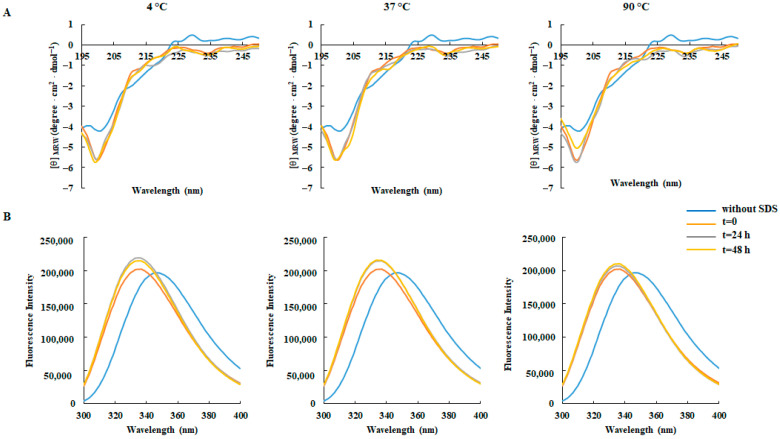
Effect of the temperature on the secondary structure and conformation of RKT1. (**A**) CD spectra and (**B**) fluorescence emission spectra of RKT1. All spectra were acquired by incubating the peptide (50 μM) in 10 mM Tris-HCl buffer, pH 7.0, in the presence of 50 mM SDS at three different temperatures after 48 h incubation.

Similar behavior was also observed when the CD and fluorescence spectra were assessed as a function of pH (Figure 3) and temperature (Figure 4), thus suggesting a high structural stability of the projected peptide under different environmental conditions.

### 3.3. Antibacterial Activity of RKT1 and RKT1 Cellulose Films

To evaluate the capacity of the newly designed peptide to counteract the growth of foodborne microorganisms, two Gram-negative (*Escherichia coli* and *Salmonella* Typhimurium) and one Gram-positive (*Listeria monocytogenes*) bacteria, isolated from meat and meat products, were chosen among the most representative pathogens often associated with food poisoning. As reported in Table 2, the minimum bactericidal concentration (MBC) values showed that RKT1 in free form had antimicrobial activity against *E. coli* and *S*. Typhimurium at relatively low concentrations (20 µM). When tested against *L. monocytogenes*, the killing concentration for RKT1 was determined to be greater than 20 µM, suggesting a reduced susceptibility of Gram-positive microorganisms towards this compound, probably related to the bacterial wall composition.

It is known that spoilage microorganism pollution is another important challenge in the food industry [40]. Therefore, antibacterial studies were also performed against two spoilage bacteria, namely *Enterococcus* spp. (Gram-positive bacteria) and *Pseudomonas koreensis* (Gram-negative bacterium), which are recognized as one of the specific spoilage organisms (SSOs) of meat and meat products. The MIC and MBC values are reported in Table 3. The results indicated that RKT1 possessed a strong bacteriostatic activity against the tested bacteria, causing a 2.93 log reduction in the growth of *P. koreensis* vs. a 2.90 log in *Enterococcus* spp. after 6 h incubation. Moreover, no bactericidal activity was observed in vitro, even at the highest peptide concentration assayed (50 µM).

Next, before estimating the effectiveness of the developed packaging prototype ‘in conditions of use’, and to ensure that the RKT1 was still in the biologically active form once adsorbed on the polymeric surfaces, the stability and the bio-preservative efficacy of the grafted peptide on CI films were investigated.

Moreover, the effects of the RKT1 cellulose films on the growth of spoilage microorganisms responsible for the deterioration of meat foods were evaluated against a monoculture of *E. coli* and *P. koreensis*. These bacteria were chosen as indicator microorganisms for in vitro assays as they are common contaminants found in beef carpaccio, which is selected as a model food system for in vivo tests. Specifically, the antibacterial properties of the CI-RKT1 materials were evaluated for 24 h of exposure, using unfunctionalized CI slides as a negative control. Overall, no significant differences in bacterial growth were observed across the functionalized and unfunctionalized materials. These results were not comparable with those obtained with RKT1 when used in free form, as reported in Table 2 and Table 3, and the lack of activity could be attributed to two factors:

(i) Immobilization can restrict the peptide’s freedom movement and flexibility, potentially forcing it into a more extended or rigid conformation compared to its solution state.
(ii) A low concentration of the RKT1 is available for interaction with the bacterial membranes once immobilized. Indeed, most of the peptide could be adsorbed primarily within the porous cellulose matrix rather than on the exposed surface directly in contact with bacterial membranes, thus rendering the films ineffective in inhibiting bacterial growth.

However, it is challenging to relate the activity of immobilized RKT1 with the performance of the free peptide since the concentration in solution and the peptide density on the surface of CI films are not directly comparable.

For these reasons, a double-conjugation strategy was set up to increase the density of peptide molecules on the cellulose surface. Briefly, RKT1 at a 50 µM concentration was adsorbed twice onto the same side of the CI disks, following the protocol optimized for the ‘one-step’ procedure. Once again, the downstream analysis confirmed the high immobilization yield (100%) (Figure S1), corresponding to a surface coverage of approximately 16.6 nmol/cm^2^.

**Figure 5:**
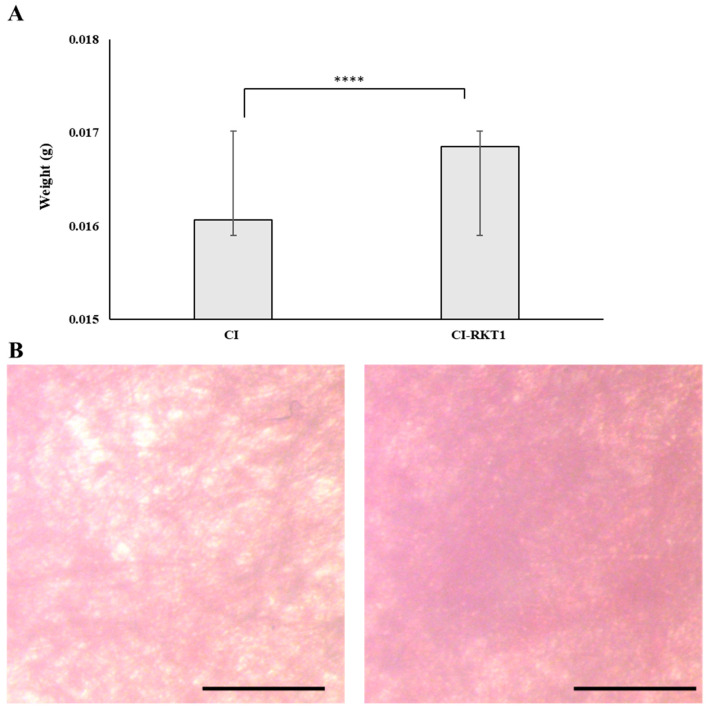
Evaluation of the absorption of RKT1 on cellulose films. (**A**) Weight variations in cellulose films before and after treatment with RKT1 solution (50 µM for each absorption step). The amount of adsorbed RKT1 on the cellulose films was determined as the difference in weight between the pristine cellulose films (CI) and the cellulose films functionalized with RKT1 (CI-RKT1). Statistical analysis was performed by comparing the experimental groups (*t*-test); **** significant difference at *p* < 0.00001. (**B**) Stereomicroscope micrographs of cellulose films untreated (**left panel**) and treated with RKT1 (**right panel**) after staining with Ponceau Red solution. Scale bar is equal to 100 μm.

To confirm the occurrence of the adsorption process, cellulose films were weighed before and after being submerged in the AMP solution (Figure 5A). The results indicated that films modified with RKT1 significantly increased their weight by 5% compared to pristine ones, strongly evidencing that the peptide was successfully adsorbed onto the cellulose. To further corroborate our results, a stereomicroscope analysis of RKT1-treated and untreated cellulose films was performed after staining with Ponceau Red, which is a simple, non-destructive method commonly used to confirm the presence and distribution of proteins on membranes. As reported in Figure 5B (left panel), a heterogeneous three-dimensional organization of the cellulose fibers and a uniform background due to specific adsorption of dye were observed on the untreated films. Conversely, a more compact and concentrated distribution of red color was observed on the RKT1 films, thus demonstrating the presence of peptide on the cellulose (Figure 5B, right panel).

**Figure 6:**
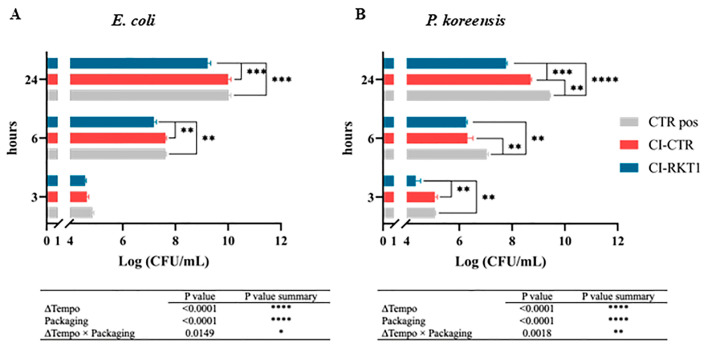
In vitro antimicrobial activity of CI-RKT1 packaging. Antimicrobial effect of CI-RKT1 was assessed on the monoculture of (**A**) *E. coli* and (**B**) *P. koreensis*. CTR pos: monoculture without any cellulose films. Results are expressed in log (CFU/mL) as mean ± error standard. Statistical analysis was performed by comparing the experimental groups at each sampling time point (Tukey’s multiple comparisons test): * significant difference at *p* < 0.05; ** significant difference at *p* < 0.01; *** significant difference at *p* < 0.001; **** significant difference at *p* < 0.0001. Additionally, an analysis of variance was performed to investigate the effect of each variable (*p* < 0.05).

Moreover, the functionalized surfaces were placed in contact with a solution of NaCl 1% for 7 days, and the release of peptide from the films over time was followed by RP-HPLC to evaluate the stability of the projected system. As evidenced in Figure S2, a scarce release (15%) of the peptide from the polymeric surfaces was observed during the first 24 h of incubation, while no further detachment was detected in the next 6 days, highlighting the high stability of the RKT1 cellulose films. Hence, the new packaging prototype was tested against the monoculture of *E. coli* and *P. koreensis*. As reported in Figure 6, CI-RKT1 exhibited antimicrobial activity toward both bacteria, causing a reduction of approximately 1 log (CFU/mL) after 24 h of incubation (*p* < 0.001). In detail, *P. koreensis* was found to be more sensitive to the active packaging (CI-RKT1) (Figure 6B) than *E. coli* (Figure 6A), showing significant differences between samples (CI-CTR vs. CI-RKT1) even after 3 h of incubation (*p* < 0.01, Tukey’s multiple comparisons test). Meanwhile, a slight antibacterial property was detected for CI-CTR only against *P. koreensis*. Statistical data relating to the analysis of variance confirmed all the described differences observed between CI-CTR and CI-RKT1 (*p* < 0.0001; two-way ANOVA). Therefore, the encouraging results prompted us to perform the tests ‘in vivo’ with the ‘new’ packaging prototype using beef carpaccio as the food model.

### 3.4. Effect of Active Packaging Prototype on Beef Carpaccio

In recent decades, consumers have become increasingly interested in fresh foods, especially those ready-to-eat foods that do not require further handling and save time. In this scenario, the correct application of good manufacturing practices and compliance with the microbiological limits set by current community legislation (Reg. CE 2073/2005), aimed at protecting consumers from pathogenic bacteria and extending the shelf life of these foods without compromising their organoleptic features, become crucial. Herein, significant advancements in the agri-food sector have been achieved with the introduction of antimicrobial packaging, offering new solutions for extending food shelf life, enhancing safety, and reducing food loss [41].

In this study, the effect of a prototype of eco-friendly active packaging on the shelf life of beef carpaccio was evaluated, considering both microbiological and physical–chemical profiles. As reported in the Materials and Methods section, two different batches of beef carpaccio were included in the experimental design to provide validated and repeatable data. This approach allowed us to define the extent to which the initial microbiological quality of beef carpaccio could influence the antimicrobial efficiency of CI-RKT1.

Specifically, microbiological results were reported in Table 4 and Table 5 for trial I and trial II, respectively. Most of the influential SSOs were investigated, choosing bacterial families of the genus of interest. *E. coli* was never found in any sample and, therefore, not included. Referring to microbiological results, significant differences in bacterial concentration were found only for *Enterobacteriaceae*, *Pseudomonas* sp., *Brochothrix* sp. and yeast in both trials, with a slight inter-batch variability.

Specifically, statistical differences were observed during the first trial between experimental groups for *Enterobacteriaceae*, with lower concentrations in beef carpaccio packaged with CI-RKT1 (cbRKT1) (*p* < 0.05; Table 4). However, in the second trial performed on another batch these differences were smaller and not significant (Table 5). Similar behavior was also evaluated for *Pseudomonas* sp. and *Brochothrix* sp., for which the effect of CI-RKT1 was found significant when analyses were performed only on batch 1 (*p* < 0.05, 2way ANOVA; Table 4). Concerning these results, two considerations are necessary: (i) the antimicrobial effect of CI-RKT1 on *Pseudomonas* genus was previously predicted with in vitro assays (Figure 6B); therefore, the microbiological data obtained on beef carpaccio are comforting; (ii) the beef carpaccio samples of batch 2 showed a better initial microbiological profile than those of batch 1, and presumably this difference affected the results. This may seem counterintuitive, but by comparing the microbiological profiles of the samples belonging to the two batches, it is possible to notice that when the starting concentration of the microorganisms was higher, the antimicrobial action of CI-RKT1 became more marked and significant.

However, as there is an exception to every rule, the antimicrobial action of CI-RKT1 against yeast, one of the main s causes of food spoilage [42], was strong, and the fungi concentration was significantly different between cbCTR and cbRKT1 samples in both trials at 7 days of storage (*p* < 0.05), as also confirmed by the analysis of variance (*p* < 0.05; Table 4 and Table 5). For this reason, the antimicrobial effect of CI-RKT1 observed in multiple tests demonstrated the prototype’s potential and its effective application in the meat sector.

Due to the differences in microorganism concentrations between the two batches (trial I and trial II), a three-way ANOVA test was performed to evaluate the effect of packaging independently of microbial contamination at the beginning of the shelf life. As reported in Table 6, the effects of the prototype of active packaging were significant on the levels of *Pseudomonas* sp., yeast, and *Brochothrix* sp. This finding provided greater consistency to the results, confirming the effectiveness of the cellulose packaging functionalized with the RKT1 peptide on the microorganisms responsible for meat spoilage.

Since the use of tightly adhering cellulose sheets on beef carpaccio can influence the water activity of the products, aw was measured throughout the test period. In this regard, no significant differences were found between the experimental groups (Figure S3).

**Figure 7:**
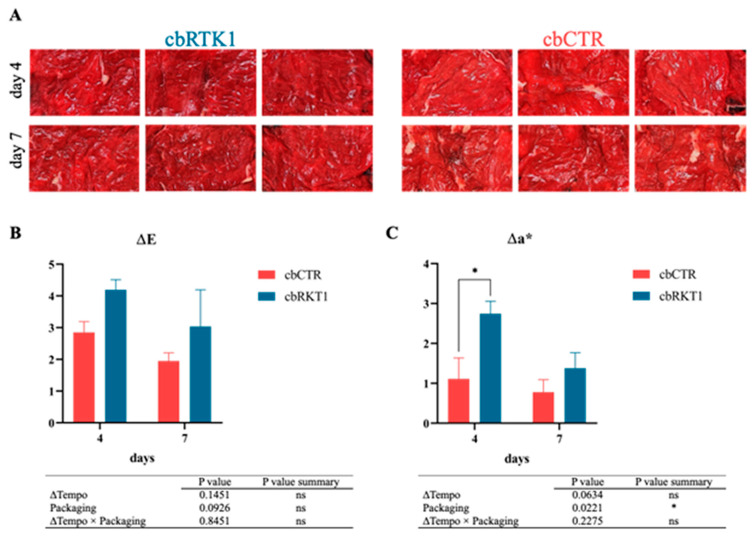
Effect of active packaging prototypes on beef carpaccio. (**A**) Macroscopical changes in pigmentation of beef carpaccio during storage at refrigerated temperature (4 ± 1 °C) for 7 days. (**B**) Total color difference (ΔE) and (**C**) variation in a* (Δa*) in beef carpaccio. cbRKT1: beef carpaccio packaged with CI films functionalized with RKT1; cbCTR: beef carpaccio packaged in non-functionalized CI films. Results are expressed as mean ± error standard. Statistical analysis was performed by comparing the experimental groups at each sampling time point (Holm-Šídák multiple comparisons test): * significant difference at *p* < 0.05. Additionally, an analysis of variance was performed to investigate the effect of each variable (*p* < 0.05).

It is necessary to emphasize that the meat spoilage cascade is characterized by discoloration, oxidative rancidity, and then microbial degradation, which occur in this order [43]. Therefore, the effect of a bound peptide on lipid oxidation and color needs to be analyzed. Although no significant differences in malondialdehyde concentration (mg/Kg) were found between the experimental groups (Figure S4), an interesting effect of CI-RKT1 on the color of beef carpaccio was observed. As shown in Figure 7A, macroscopic differences were already evidenced on day 4 of storage, with a discoloration of cbCTR samples. This should not be underestimated, being aware that consumers are strongly influenced by the color of the meat when purchasing. Therefore, to easily describe the total chromatic variation and the loss of the typical red color of fresh beef (discoloration), ΔE and Δa* were calculated. According to Equation (1), all the coordinates (L*, a*, and b*) are considered for the estimation of ΔE, reflecting the changes in the pigmentations of meat tissue, while the second index, Δa*, specifically represents the variability of the redness. In detail, no significant differences were observed between cbCTR and cbRKT1 for the ΔE index (Figure 7B), which was slightly higher in cbRKT1 samples due to both the weak brightness loss of these samples and higher a* values (Table S1). Instead, interesting data in terms of redness variation and loss of the intense red color were obtained from the calculation of Δa* (Figure 7C), which was found to be lower in cbCTR samples (*p* < 0.05, two-way ANOVA).

To sum up, the results can be considered satisfactory, allowing us to demonstrate that the organoleptic aspect of the carpaccio was not negatively influenced since CI-RKT1 seems to increase the red color of carpaccio beef, which is known to encourage consumer purchase and consumption. Nevertheless, further studies are needed to state that the use of the active packaging prototype enhances color.

In an era prioritizing environmental impact, unnecessary wastage, and reduction in costs, designing reusable packaging becomes crucial, as it significantly reduces environmental burdens compared to single-use alternatives. Therefore, to assess whether the projected packaging could be reused without compromising its effectiveness, the in vivo experiments were also conducted using the same films multiple times, after abundant washing with water and drying at room temperature. Interestingly, the RKT1 cellulose surfaces were reused successfully at least six times without loss of functional activity, thus representing an important advantage from the industrial point of view and making the technology more cost-effective and sustainable.

## 4. Conclusions

With the ever-increasing demand for minimally processed food products, innovative active packaging is evolving in this era [44,45,46]. However, the development of biodegradable and environmentally friendly layers with long-lasting antibacterial activities and relatively low costs remains an unsolved problem, requiring further investigations. In this context, the results from this pilot study provide a feasible approach to develop antimicrobial cellulose-based materials with potential applications as active food packages. Partial immobilization of the newly designed peptide RKT1 onto the surface of cellulose papers was realized by using a green, simple, and efficient preparation strategy, mainly exploiting the electrostatic interactions between AMP and polymeric films. The antimicrobial activity of RKT1-loaded paper sheets was then assessed, demonstrating that the peptide remained active once immobilized, and films have great potential as a support for creating antimicrobial materials to preserve meat samples, extending shelf life and ensuring their safety. However, more in-depth studies will be necessary to confirm the suitability of the new projected system as a viable alternative to conventional plastics, focusing on its effectiveness, sustainability, and feasibility in real-world scenarios.

## Figures and Tables

**Table 1:** Physicochemical properties of the peptide RKT1.

Parameters	RKT1
Sequence	RKTWLRIWKR-NH_2_
Molecular weight (Da)	1442.78
Bomax index (kcal/mol)	4.39
Net charge	+5
Half-life (sec)	952.81
Hydrophobicity	−0.57
Hydropathicity	−1.55
Amphipathicity	1.47
Hydrophilicity	0.42

**Table 2:** MBC values of RKT1 against pathogenic bacteria.

Pathogenic Bacteria	MBC (µM)
*E. coli*	20
*L. monocytogenes*	20
*S. Typhimurium*	40

**Table 3:** MIC and MBC values of RKT1 against spoilage bacteria.

Pathogenic Bacteria	MIC (µM)	MBC (µM)
*P. koreensis*	10.0 ± 1.6	>50
*Enterococcus* spp.	12.5 ± 2.5	>50

**Table 4:** Microbiological counts [log (CFU/g)] in beef carpaccio samples belonging to batch 1 packaged with CI-RKT1.

Trial I		Days			Effect (*p* Value)		
		d0	d4	d7	ΔTempo	Packaging	ΔTempo × Packaging
*Enterobacteriaceae*	cbCTR	1.26 ± 0.07	1.20 ± 0.24	1.44 ± 0.48	0.4655	0.0730	0.9880
	cbRKT1		ni	0.73 ± 0.23			
			*	*			
*Pseudomonas* sp.	cbCTR	5.66 ± 0.23	5.71 ± 0.04	8.56 ± 0.12	0.0002	0.0186	0.9305
	cbRKT1		4.87 ± 0.43	7.62 ± 0.22			
				*	***	*	
TMB	cbCTR	6.10 ± 0.45	4.91 ± 0.05	7.89 ± 0.11	<0.0001	0.0868	0.6011
	cbRKT1		4.46 ± 0.20	7.63 ± 0.22			
					****		
Yeast	cbCTR	2.21 ± 0.14	2.50 ± 0.24	5.09 ± 0.05	0.0030	0.0490	0.2053
	cbRKT1		2.08 ± 0.58	3.73 ± 0.23			
				*	**	*	
Mold	cbCTR	4.14 ± 0.35	3.73 ± 0.01	5.59 ± 0.12	0.0003	0.2858	0.7121
	cbRKT1		3.59 ± 0.07	5.32 ± 0.28			
					***		
*Brochothrix* sp.	cbCTR	5.04 ± 0.20	4.50 ± 0.06	6.71 ± 0.05	0.0002	0.0289	0.7468
	cbRKT1		3.85 ± 0.11	6.18 ± 0.33			
					***	*	
LAB	cbCTR	3.97 ± 0.21	3.32 ± 0.36	5.11 ± 0.08	0.0025	0.7860	0.6479
	cbRKT1		3.51 ± 0.02	5.06 ± 0.33			
					**		
Coagulase-positive *staphylococci*	cbCTR	2.26 ± 0.14	3.10 ± 0.06	2.91 ± 0.65	0.7806	0.8548	0.8548
	cbRKT1		2.95 ± 0.21	2.91 ± 0.35			

ni: concentration lower than the minimum sensitivity limit of the ISO method. Results are expressed in log (CFU/g) as mean ± standard error of three different replicates. Statistical analysis was performed by comparing the experimental groups at each sampling time point (Holm-Šídák multiple comparisons test): * significant difference at *p* < 0.05; ** significant difference at *p* < 0.01; *** significant difference at *p* < 0.001; **** significant difference at *p* < 0.0001. Additionally, an analysis of variance was performed to investigate the effect of each variable (*p* < 0.05).

**Table 5:** Microbiological counts [log (CFU/g)] in beef carpaccio samples belonging to batch 2 packaged with CI-RKT1.

Trial II		Days			Effect (*p* Value)		
		d0	d4	d7	ΔTempo	Packaging	ΔTempo × Packaging
*Enterobacteriaceae*	cbCTR	1.44 ± 0.12	1.65 ± 0.21	1.31 ± 0.35	0.1766	0.8812	0.5008
	cbRKT1		1.87 ± 0.43	0.97 ± 0.47			
*Pseudomonas* sp.	cbCTR	3.04 ± 0.54	4.68 ± 0.42	7.44 ± 0.44	0.0025	0.3084	0.4920
	cbRKT1		4.53 ± 0.27	6.74 ± 0.30			
				*	**		
TMB	cbCTR	3.65 ± 0.41	4.53 ± 0.27	6.20 ± 0.36	0.0019	0.5873	0.8041
	cbRKT1		4.61 ± 0.05	6.41 ± 0.15			
					**		
Yeast	cbCTR	ni	2.44 ± 0	4.26 ± 0.30	0.0018	0.0125	0.7529
	cbRKT1		ni	3.17 ± 0.36			
				*	**	*	
Mold	cbCTR	2.86 ± 0.37	3.39 ± 0.39	4.83 ± 0.17	0.0024	0.0849	0.6826
	cbRKT1		3.81 ± 0.01	5.44 ± 0.14			
					**		
*Brochothrix* sp.	cbCTR	2.56 ± 0.20	3.97 ± 0.26	5.03 ± 0.46	0.0160	0.1761	0.9208
	cbRKT1		3.49 ± 0.09	4.61 ± 0.05			
					*		
LAB	cbCTR	2.26 ± 0.10	2.94 ± 0.20	2.50 ± 1.00	0.9036	0.6474	0.5297
	cbRKT1		2.83 ± 0.27	3.13 ± 0.13			
							
Coagulase-positive *staphylococci*	cbCTR	ni	2.83 ± 0.57	2.77 ± 0.03	0.7451	0.1920	0.8518
	cbRKT1		2.26 ± 0.30	2.03 ± 0.53			

ni: concentration lower than the minimum sensitivity limit of the ISO method. Results are expressed in log (CFU/g) as mean ± standard error of three different replicates. Statistical analysis was performed by comparing the experimental groups at each sampling time point (Holm-Šídák multiple comparisons test): * significant difference at *p* < 0.05; ** significant difference at *p* < 0.01. Additionally, an analysis of variance was performed to investigate the effect of each variable (*p* < 0.05).

**Table 6:** Three-way analysis of variance (ANOVA) highlighting the main effects and interactions between ANOVA factors. Results showing *F*-values and significance level of effects and their interaction on the concentrations of each microorganism studied.

Value		ΔTempo (ΔT)	Batch (B)	Packaging (P)	ΔT × B	ΔT × P	B × P	ΔT × B × P
*Enterobacteriaceae*	*F*	0.8664	3.2960	3.660	2.5820	0.4693	1.4550	0.2680
	*p*	0.4047	0.1436	0.1405	0.1834	0.5309	0.2942	0.6320
*Pseudomonas* sp.	*F*	180.90	13.50	10.75	0.5333	0.5716	0.8885	0.3040
	*p*	**0.0002 ^a^**	**0.0213**	**0.0305**	0.5057	0.4917	0.3993	0.6107
TMB	*F*	287.40	29.01	0.6031	21.18	0.2942	2.9740	0.0088
	*p*	**<0.0001**	**0.0057**	0.4808	**0.0100**	0.6163	0.1597	0.9297
Yeast	*F*	73.24	8.0370	18.46	1.0030	1.6280	0.0860	0.3023
	*p*	**0.0010**	**0.0471**	**0.0127**	0.3732	0.2710	0.7840	0.3023
Mold	*F*	258.50	1.3400	2.2520	0.5987	0.0318	4.7500	0.2410
	*p*	**<0.0001**	0.3115	0.2078	0.4823	0.8672	0.0948	0.6492
*Brochothrix* sp.	*F*	126.90	35.38	12.06	11.57	0.0908	0.1731	0.0087
	*p*	**0.0004**	**0.0040**	**0.0255**	**0.0272**	0.7781	0.6987	0.9302
LAB	*F*	8.7050	20.00	0.3793	7.6860	0.2018	0.0927	0.6037
	*p*	**0.0419**	**0.0111**	0.5713	0.0502	0.6765	0.7760	0.4806
Coagulase-positive *staphylococci*	*F*	0.1911	3.3460	1.5050	0.0033	0.0002	1.1560	0.0862
	*p*	0.6846	0.1414	0.2872	0.9570	0.9897	0.3428	0.7837

^a^ Significant *p* values are shown in bold fonts.

## Data Availability

The original contributions presented in this study are included in the article/Supplementary Materials. Further inquiries can be directed to the corresponding authors.

## Supplementary Materials

The following supporting information can be downloaded at: https://www.mdpi.com/article/10.3390/foods14152607/s1, Figure S1: Immobilization yield (%) of RKT1 on CI films (CI-RKT1) determined by RP-HPLC chromatography; Figure S2: Stability in saline solution of CI-RKT1 films determined by RP-HPLC; Figure S3: Effects of CI-RKT1 surfaces on the physical quality on beef carpaccio; Figure S4: Effects of CI-RKT1 surfaces on the chemical quality of beef carpaccio; Table S1: Changes in color indices of the beef carpaccio packaged in CI films functionalized with RKT1 during storage at 4 °C.
