# Supplementary material for: Surface Functionalization of Cellulose-Based Packaging with a New Antimicrobial Decapeptide: A Sustainable Solution to Improve the Quality of Meat Products
Source: Foods. 2025 Jul 24;14(15):2607. doi: 10.3390/foods14152607 (PMC12346316; doi:10.3390/foods14152607)
Supplement: Supplementary file 1 [file foods-14-02607-s001.zip › Figure S1.pdf]

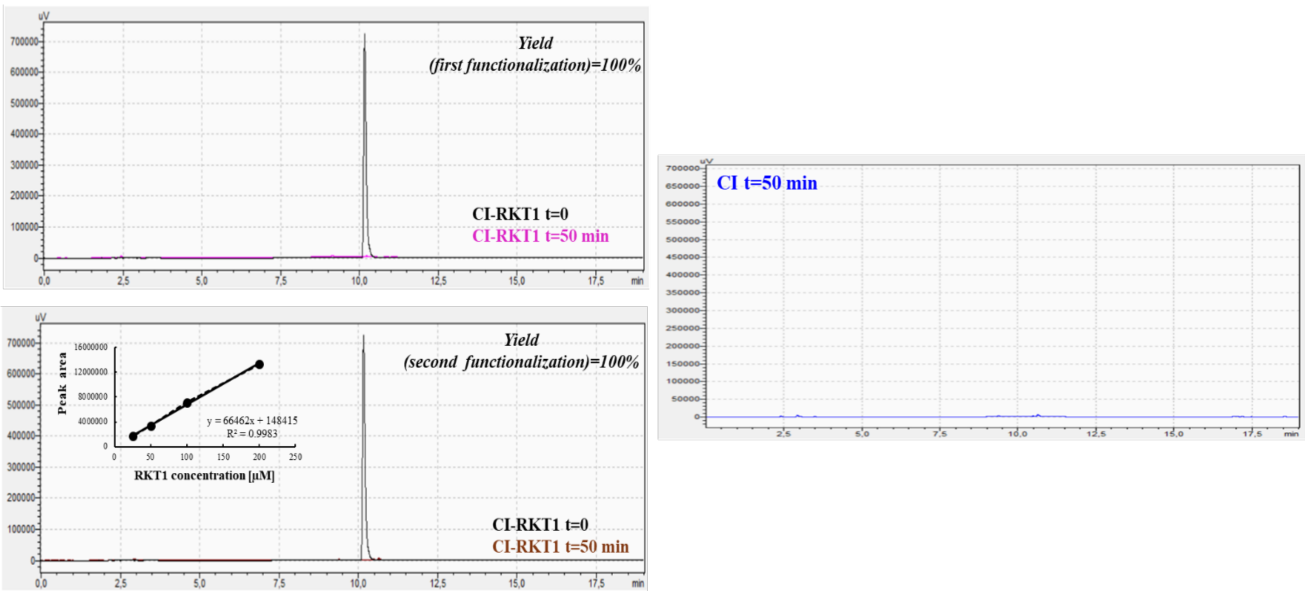


**Figure S1. Immobilization yield (%) of RKT1 on CI films (CI-RKT1) determined by RP-HPLC chromatography.** CI surfaces were incubated twice at 60 °C for 50 min with RKT1 solution (50 µM). After each incubation reaction, the solutions were recovered and analysed by RP-HPLC on a C18 column. The peptide solution placed in contact with the CI films at time 0 ($t$ = 0), and the CI slides not functionalized with the peptide were used as controls. ***Insert***: Calibration curve of C18 column obtained using different RKT1 concentrations. The chromatograms are representative of three independent experiments.
