# Supplementary material for: Surface Functionalization of Cellulose-Based Packaging with a New Antimicrobial Decapeptide: A Sustainable Solution to Improve the Quality of Meat Products
Source: Foods. 2025 Jul 24;14(15):2607. doi: 10.3390/foods14152607 (PMC12346316; doi:10.3390/foods14152607)
Supplement: Supplementary file 1 [file foods-14-02607-s001.zip › Figure S2.pdf]

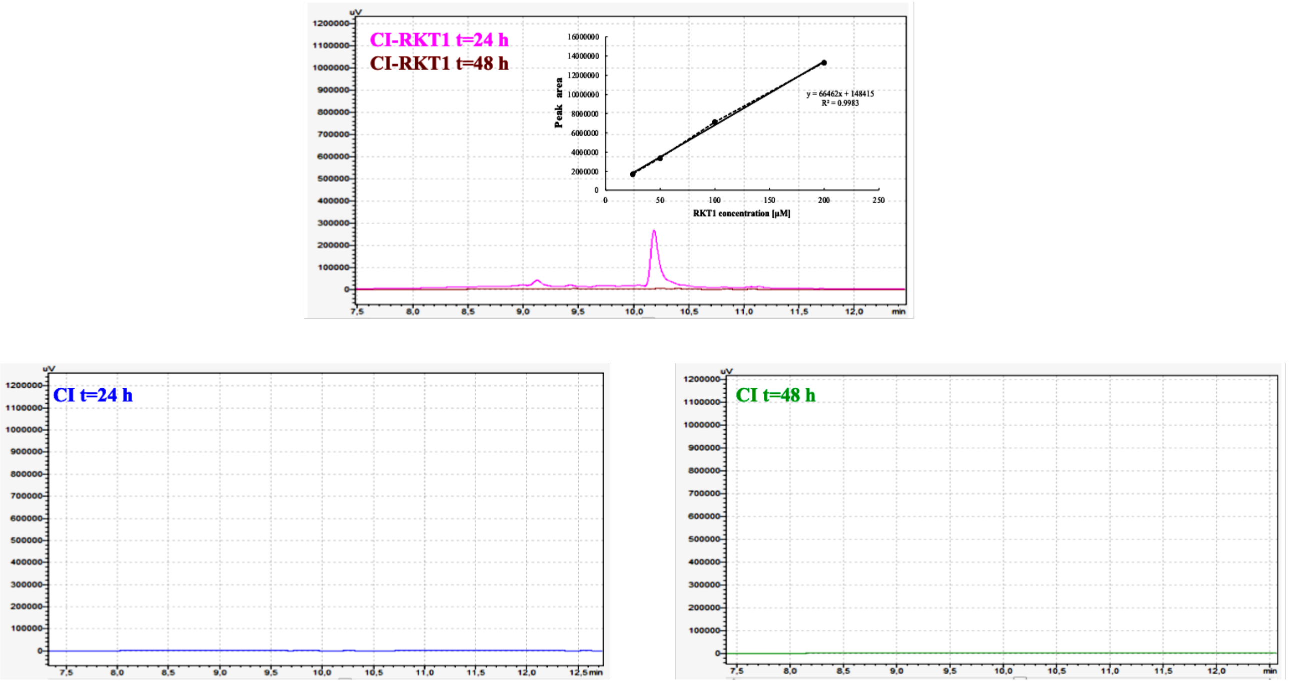

Figure S2. Release analysis of RKT1 from CI films performed by RP-HPLC. CI-RKT1 films were incubated in the presence of NaCl (1%) for 7 days at 25 °C. At each incubation time, aliquots (200 µL) were recovered and analysed by RP-HPLC on a C18 column. The CI films not functionalized with the peptide were used as controls. The percentage of release was determined by using the calibration curve (*insert*) with respect to the nmol/cm^2 of the adsorbed peptide. The chromatograms are representative of three independent experiments.
