# Supplementary material for: Surface Functionalization of Cellulose-Based Packaging with a New Antimicrobial Decapeptide: A Sustainable Solution to Improve the Quality of Meat Products
Source: Foods. 2025 Jul 24;14(15):2607. doi: 10.3390/foods14152607 (PMC12346316; doi:10.3390/foods14152607)
Supplement: Supplementary file 1 [file foods-14-02607-s001.zip › Figure S3.pdf]

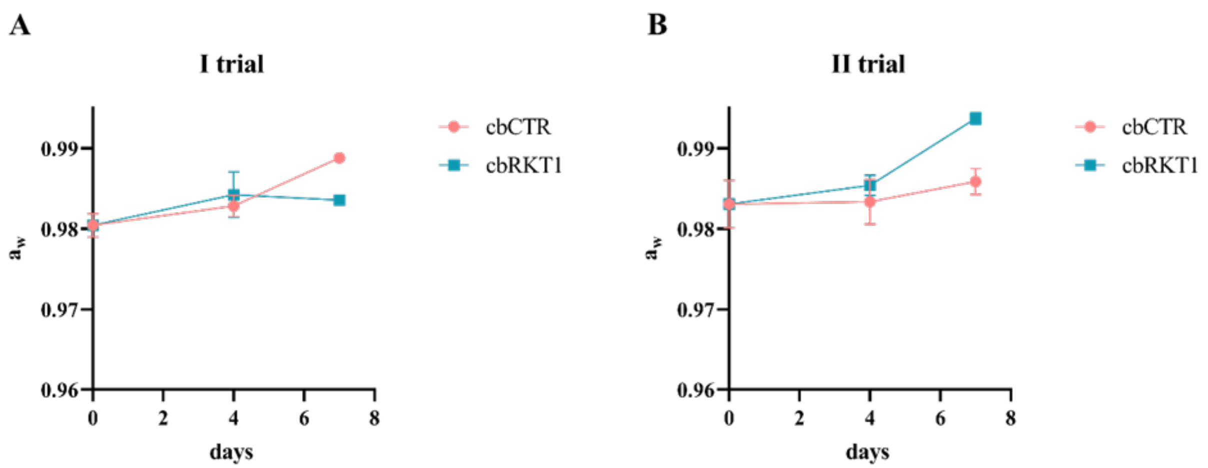


**Figure S3. Effects of CI-RKT1 surfaces on the physical quality on beef carpaccio.** Analyses of activity water (aw) in beef carpaccio samples belonged to (**A**) batch 1 and (**B**) batch 2. cbRKT1: beef carpaccio packaged with CI films functionalized with RKT1; cbCTR: beef carpaccio packaged in not functionalized CI films. Results are expressed as mean ± error standard. Statistical analysis was performed by comparing the experimental groups at each sampling time point (Holm-Šídák multiple comparisons test): no significant differences were found. No significant results were obtained from analysis of variance (2way ANOVA).
