# Supplementary material for: Surface Functionalization of Cellulose-Based Packaging with a New Antimicrobial Decapeptide: A Sustainable Solution to Improve the Quality of Meat Products
Source: Foods. 2025 Jul 24;14(15):2607. doi: 10.3390/foods14152607 (PMC12346316; doi:10.3390/foods14152607)
Supplement: Supplementary file 1 [file foods-14-02607-s001.zip › Figure S4.pdf]

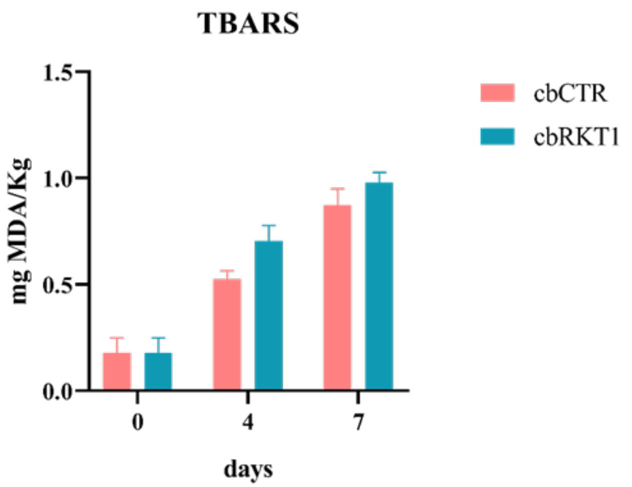

Figure S4. Effects of CI-RKT1 surfaces on the chemical quality of beef carpaccio. Measurements of thiobarbituric acid reactive substances (TBARS) expressed as malonyldialdehyde (MDA) concentration (mg/Kg) in beef carpaccio samples. Results are expressed as mean ± error standard of values obtained by analyzing both batch 1 and batch 2 of beef carpaccio. Statistical analysis was performed by comparing the experimental groups at each sampling time point (Holm-Šídák multiple comparisons test): no significant differences were found. Also, no significant results were obtained from analysis of variance (2way ANOVA).
