# Supplementary material for: Surface Functionalization of Cellulose-Based Packaging with a New Antimicrobial Decapeptide: A Sustainable Solution to Improve the Quality of Meat Products
Source: Foods. 2025 Jul 24;14(15):2607. doi: 10.3390/foods14152607 (PMC12346316; doi:10.3390/foods14152607)
Supplement: Supplementary file 1 [file foods-14-02607-s001.zip › Table S1.pdf]

Table S1. Changes in colour indices of the beef carpaccio packaged in CI films functionalized with RKT1 during storage at 4 °C.

		**Days**			**Effect (*p* value)**		
sci		d0	d4	d7	ΔTempo	Packaging	ΔTempo × Packaging
*L**	cbCTR	41.24±1.11	41.14±1.2	41.42±0.17	ns	ns	ns
	cbRKT1		40.12±0.91	40.57±1.13			
*a**	cbCTR	16.06±0.28	17.84±0.72	16.84±0.24	***	*	ns
	cbRKT1		18.81±0.59*	16.78±0.74	0.0003	0.0185	
*b**	cbCTR	14.06±0.80	15.11±0.71	13.42±0.59	ns	ns	ns
	cbRKT1		14.73±0.18	12.43±0.99			

sce							
*L**	cbCTR	38.51±1.25	38.06±1.23	38.15±0.19	ns	ns	ns
	cbRKT1		37.26±0.98	36.79±0.91			
*a**	cbCTR	16.19±0.24	18.49±0.70	16.63±0.35	***	*	ns
	cbRKT1		19.09±0.68	16.8±0.58	0.0001	0.0286	
*b**	cbCTR	18.52±0.58	19.99±0.58	19.49±0.69	ns	ns	ns
	cbRKT1		19.91±0.26	18.88±0.65			

For each coordinate, the results were reported as the mean ± standard error (sem) of the collected measurement values, including or excluding the specular component (*sci* or *sce* mode). Three samples per experimental group were analyzed per sampling day. Statistical analysis was performed by comparing the experimental groups at each sampling time point (Tukey's multiple comparisons test): * significant difference at $p < 0.05$. Additionally, an analysis of variance was performed to investigate the effect of each variable ($p < 0.05$).
